# Misunderstood and underappreciated: a critical review of mental health advocacy and activism in low- and middle-income countries

**DOI:** 10.1093/heapol/czae016

**Published:** 2024-03-05

**Authors:** Alma Ionescu, Jenevieve Mannell, Megan Vaughan, Rochelle Burgess

**Affiliations:** Institute for Global Health, University College London, 30 Guilford Street, London WC1N 1EH, United Kingdom; Institute for Global Health, University College London, 30 Guilford Street, London WC1N 1EH, United Kingdom; Institute for Advanced Studies, University College London, South Wing, Wilkins Building, Gower Street, London WC1E 6BT, United Kingdom; Institute for Global Health, University College London, 30 Guilford Street, London WC1N 1EH, United Kingdom

**Keywords:** Mental health, advocacy, review, developing countries, context, social movements

## Abstract

Mental health advocacy and activism have been highlighted as important in the effort towards creating environments for better mental health. However, relevant research in low- and middle-income country settings remains limited and lacks critical exploration. We seek to contribute to filling this gap by exploring driving factors behind mental health advocacy and activism efforts in low- and middle-income country settings. This review uses a critically informed thematic analysis employing conceptual frameworks of productive power to analyse peer-reviewed articles on mental health advocacy or activism over the last 20 years. We suggest that the current body of research is marred by superficial explorations of activism and advocacy, partly due to a lack of cohesion around definitions. Based on our findings, we suggest a conceptual framework to guide deeper explorations of mental health advocacy and activism. This framework identifies ‘legitimacy’, ‘context’ and ‘timing’ as the main dimensions to consider in understanding activism and advocacy efforts. The fact that they remain misunderstood and underappreciated creates missed opportunities for meaningful inclusion of lived experience in policy decisions and limits our understanding of how communities envision and enact change.

Key messages

- The literature on mental health activism and advocacy in low- and middle-income country settings is growing but still fragmented, with no cohesive or common operationalization of the definitions of activism or advocacy.
- The lack of a commonly accepted definition or understanding allows for the co-optation of activism and advocacy to the advantage of external actors or stakeholders.
- Analysis of the dimensions of legitimacy, context and timing (our proposed framework) can help to clarify how meaning-making and goals of mental health advocacy and activism are constructed, and therefore help shed light on the potential value and importance of mental health advocacy and activism to the various stages of policy-making.

## Introduction

The momentum of the Global Mental Health (GMH) movement has continued at pace, particularly since the publication of the Lancet Commission which highlighted the importance of mental health to wider development processes (Patel and Prince, 2010; Collins *et al*., 2011). Delivering service equity is a key driver of the movement and underpins many innovations prioritized by the movement (Patel *et al*., 2011; Kirmayer and Pedersen, 2014). In an effort to strive towards equity, the importance of centring patient voices and communities within GMH has been put forward as a crucial aspect (Campbell and Burgess, 2012). Indeed, the central principle (borrowed from the disability movement)—nothing about us without us*—*makes this very clear (Patel *et al*., 2018). An important pathway for listening to community voices is through advocacy and activism—as they seek to centre those very voices.

Health activism is broadly defined as challenging power relationships and existing orders where those are felt to negatively affect aspects of health and health promotion (Zoller, 2005). As such, activism is more directly defined by its aims, namely creating health-enabling social environments, than by its methods. This is reflected in a growing body of literature that draws our attention to other methods and forms of activism, such as quiet or everyday activism, and challenges the public image that is restricted to associations with social movements and protests (Chatterton and Pickerill, 2010; Pottinger, 2017; Rose, 2018; Campbell and Cornish, 2021). In particular, critical public-health activism emphasizes that collective action is advanced by vulnerable or marginalized groups and their allies in an effort to redistribute power (Campbell, 2020; Campbell and Cornish, 2021). In line with these understandings and definitions of critical health activism, our review chooses to focus on advocacy and activism efforts that are community- and service-user led. We are therefore omitting forms of advocacy or activism nested within the broader landscape that may be more centralized or top-down in nature, such as global networks (Shiffman *et al*., 2016).

In the context of mental health activism and advocacy specifically, unequal power relations within the mental health industrial complex form a particular point of tension that advocates have sought to address. This is why service users and communities have been at the forefront of driving the push for change. Some of the literature particularly highlights the important role of advocacy in light of the unequal power relations and epistemic injustices patients face (Newbigging *et al*., 2015; Ridley *et al*., 2018). Patient voices have traditionally been silenced in these spaces, which has been detrimental both to the individual’s personal recovery as well as to our knowledge base. The omission of lived experience constricts the realm of our possibilities for knowledge in relation to mental health by discrediting the value of experiences and subjectivity (Crossley, 2006; Molas, 2016; Beresford, 2020). Molas (2016) therefore describes advocacy as being (positively) disruptive as it foregrounds voices that are marginalized by and within these wider systems and structures. This makes advocacy a critical component of comprehensive mental health systems, ensuring both the protection of patient rights and the best health outcomes (Stylianos and Kehyayan, 2012).

### Mental health advocacy and activism—in search of meaning

Although agreement on the importance of mental health-related advocacy and activism exists, there is acknowledgement that a coherent definition is lacking and underlying processes and mechanisms remain elusive (Perry, 2013; Stomski *et al*., 2017). Similarly, the theoretical foundations and conceptualizations of its impact are poor. The evidence base is weak as the efforts of organizations (e.g. such as non-governmental organisations) engaging in advocacy and empowerment remain largely understudied and unexamined (Boyle *et al*., 2007). For example, despite decades of advocacy activities in Australia, Morrison *et al*. (2018) note that they were able to only identify two studies that explored mental health advocacy at the time of writing. The resulting lack of rigour around operationalization of definitions creates a considerable impediment for advocacy to receive the rightful attention it deserves. This has been observed outside academic circles too, as confusion around mental health advocacy and activism has also been observed across stakeholder groups such as patients, mental health professionals, social workers and more (Newbigging *et al*., 2015; Maylea *et al*., 2020). This problem is only further compounded by the fact that advocacy and activism are words that are in many ways socially loaded—some seeing them as ‘dirty words’ (Parsons, 2016), which creates tendencies within research to steer away from them, to the detriment of policy and practice. These gaps—both academic and non-academic—point to the need for more research to solidify our foundational knowledge about processes of activism and advocacy in mental health spaces around the world.

### The need to learn from low- and middle-income countries

A lack of research documentation on what successful efforts look like has been particularly noted within low- and middle-income country (LMIC) settings (Hann *et al*., 2015). In light of the comparably larger volume of literature on advocacy and activism in high-income (HIC) settings, there might be a temptation to transfer the meanings and understandings to LMIC settings. Whilst there is much we can learn from those movements (Trivedi, 2014), we believe that there is a need for inquiry into the meanings of advocacy and activism from LMICs themselves.

It is at this junction that our review seeks to make its contribution. There is a lack of a systematic understanding of mental health advocacy and activism in LMICs, which widens our knowledge gaps and therefore narrows the possibilities for evidence-informed action. Our review seeks to create an overview and critically interrogate the discourse around this topic by exploring the following question: What factors drive (or inhibit) mental health advocacy and activism efforts in LMICs?

## Methods

The format of this review follows that of a critical review, as outlined by Grant and Booth (2009). Whilst still an emerging form of reviewing literature, it is growing in use and has recently been employed to critically examine meanings around community in the GMH space (Elias *et al*., 2021) One of its strengths lies in the ability to identify and discuss areas that need strengthening in the given field of research (Paré *et al*., 2015), which lends itself well to the aim of the review. It provides an opportunity to highlight contradictions, limitations and inconsistencies (Elias *et al*., 2021). By evaluating these, we are able to ‘take stock’ and consequently evaluate the aspects of value in the current body of work (Grant and Booth, 2009). The resulting conceptual contribution is presented in the form of a model and suggestions for future research on this topic.

### Inclusion and exclusion criteria

We included both primary and secondary research, as well as papers reporting both qualitative and quantitative methods. We chose to include papers published between the years 2001 and 2023, which aligns with the publication of the Wolrd Health Organisation (WHO) World Health Report on Mental Health (World Health Organisation, 2001), commonly considered as the launch of global efforts to increase the quality of and access to mental health services. We included papers from LMICs, as per the classification of the World Bank. With the help of the subject library, we adapted the Cochrane EPOC LMIC geographical filter to capture relevant papers in our database search. We excluded papers that used the verb ‘advocate’ in ways that were not underpinned by the notion of social change or taking action, but instead merely used it as a passive verb to denote ‘being in agreement’ with an issue. As ‘advocacy’ and ‘activism’ are often used interchangeably and fluidly (McKeever *et al*., 2023), we chose to include papers looking at both in order not to omit relevant publications. We included papers published in English. Table 1 provides an overview of the inclusion and exclusion criteria.

**Table 1.** Exclusion and inclusion criteria

**Exclusion criteria**	**Inclusion criteria**
Articles without a central focus on mental health Mental health discussed in direct relation to another issue (e.g. HIV)	Focused on mental health Low- and middle-income countries settings Activism/advocacy not required to be the sole focus of the paper Primary and secondary research Qualitative and quantitative research English language publication Publication in peer-reviewed journals Published between 2001 and 2023

### Information sources

**Figure 1.**
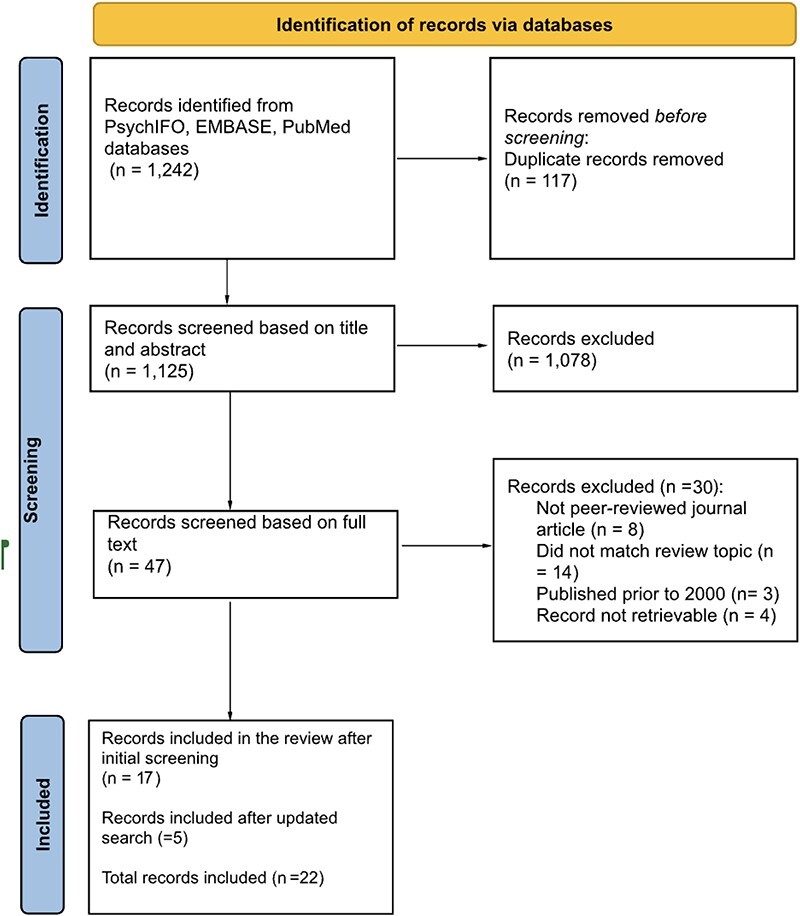
PRISMA flow diagram

Searches were conducted on PsychINFO, Embase and PubMed in December 2021. We worked with a subject librarian to define the search terms. The searches performed included a string of terms related to mental health, advocacy, activism, user organizations and movements. The initial searches provided 1242 results. A total of 117 duplicates were discarded, which led to a total of 1125 records. An initial round of screening was performed by author AI based on title and abstract using the inclusion and exclusion criteria. This left us with 47 papers. A second screening was performed by AI based on full texts using the same criteria. Papers were excluded if they were not peer-reviewed, if the full paper did not match the topic of the review, if they were published prior to 2001 or if full-text was not retrievable. After the screening process, 17 papers remained. An updated search was conducted in November 2023, which yielded an additional 5 papers for inclusion, bringing the final number of papers included in the review to 22. The screening process is presented in Figure 1. Additionally, we included texts, such as reports, from three organizations (Mariwala Health Initiative in India, Kushinga in Zimbabwe, Talang Dalisay in the Philippines), identified through online searches, as grey literature to supplement the analysis. We identified organizations that covered various geographical locations and central foci. We did however not undertake a systematic review of the grey literature, as we were interested in interrogating the discourse around advocacy and activism and how it is framed as academic evidence.

We compiled a document of key characteristics of the papers into an Excel spreadsheet (Table 2). This enabled us to have a clear overview of the papers, particularly in terms of whether they had a central focus on advocacy and activism or not.

**Table 2.** Data extraction table for included paper characteristics

Authors	Qualitative/quantitative	Methods	Central focus	Topic	Main participants	Country
Abayneh *et al*. (2017)	Qualitative	Semi-structured interviews	No	Assessing possibilities of service-user involvement	Service users	Ethiopia
Abdulmalik *et al*. (2014)	Qualitative	Secondary data	Yes	MH Leadership and advocacy programme	not specificed	West Africa
Agyapong *et al*. (2019)	Mixed methods	Survey questionnaires and FGD	No	Promotion of psychiatry through public speaking competition	Medical students	Ghana
Anbessie *et al*. (2023)	Qualitative	Descriptive	Yes	Role of media in mental health advocacy	not specified	Ethiopia
Ardila-Gómez *et al*. (2020)	Qualitative	Interviews, participatory observations, document analysis	Yes	History and analysis of user movement	Key informants	Argentina
Asante *et al*. (2021)	Qualitative	Semi-structured interviews	No	Role of NGOs in supporting recovery	NGO staff	Ghana
Davies *et al*. (2021)	Mixed methods	Semi-structured interviews	Yes	Evaluation of national advocacy programme	Various stakeholders	South Africa
Deshpande *et al*. (2013)	Qualitative	Secondary data	No	Response to new MH legislation	not specified	Egypt and India
Fitts *et al*. (2020)	Qualitative	Semi-structured interviews	No	Strengthening mental health systems	Various stakeholders	Sierra Leone
Hann *et al*. (2015)	Qualitative	FDGs and key-informant interviews	Yes	Factors for success in MH advocacy	Stakeholders and advocacy targets	Sierra Leone
Hendler *et al*. (2016)	Qualitative	Semi-structured interview	Yes	Exploring the practice and promise of advocacy	Leaders in health and mental health	Zimbabwe
Irmansyah *et al*. (2020)	Qualitative	Interviews	Yes	Stakeholder perceptions on civic involvement	Health professionals and other stakeholders	Indonesia
Kimangale *et al*. (2021)	Qualitative	Secondary data	No	Challenges and opportunities for psychology	not specified	Tanzania
Kitafuna (2022)	Qualitative	Descriptive	Yes	Mental health system and activism	not specified	Uganda
Kleintjes *et al*. (2012)	Qualitative	Semi-structured interviews	No	Views of service users on improving recovery	Service users	South Africa
Mohamed (2015)	Qualitative	Descriptive	No	Reviving the MH policy	n/a	Maldives
Nickels *et al*. (2016)	Qualitative	Focus-group discussions, questionnaire	No	Family MH self-help programmes	Users and caregivers	El Salvador
Rose (2018)	Qualitative	Interviews, participant observation	Yes	Effects and meaning of participation in user groups	MH user association members	Argentina
Sorsdahl *et al* (2012)	Quantitative	Survey	Yes	Internalized stigma in user-group members	MH user association members	South Africa
Ssebunnya *et al*. (2018)	Mixed methods	Key-informant interview and document analysis	No	Sustainable MH financing	not specified	Uganda
Stein and Seedat (2007)	Qualitative	Descriptive	No	Challenges and opportunities in psychiatry	not specified	‘Developing world’
Upadhaya *et al*. (2014)	Qualitative	Descriptive	No	The role of MH NGOs	not specified	Nepal

The critical review format does not require a quality appraisal, given its primary concern with taking stock of a particular body of knowledge rather than to systematically assess a wider body of evidence (Grant and Booth, 2009), and as such, the decision was made to not undertake a quality appraisal. However, all papers included in the study are peer-reviewed.

### Analysis and data extraction

The analysis was undertaken using a critically informed thematic analysis. While the data extraction was data-driven, we analysed the data using a conceptual framework informed by theories of productive power. Our choice for a critical approach was made in awareness of calls to analyse unequal power relations in global health (Shiffman, 2014). Our reading of the papers was informed by theories of productive power. Productive power is defined as concerning itself with discourses, social processes and systems of knowledge through which meaning is created (Barnett and Duvall, 2005). Productive power affects how we see the world and ourselves in it. For this reason, we decided to also include papers that did not have a central focus on activism and advocacy, but that did have a dedicated section on advocacy or activism. This allowed for a more well-rounded interrogation around advocacy and activism as it includes different perspectives that contribute to shaping our understanding in different ways.

We followed a six-step approach as outlined by Braun and Clarke (2006). In the first instance we familiarized ourselves with the data, followed by extracting data in terms of key characteristics (found in Table 2), such as methods, context/setting and whether they had a central focus or not. Following this, initial codes were then compiled into emerging themes. We then reviewed the themes, and subsequently named the themes. We drew on Burgess and colleagues’ (Burgess *et al*., 2022) operationalization of thematic analysis, which adds higher-order theme ‘thematic categories’, which enables the ability to bring together various themes and categories in the analysis. In the last step of writing-up and producing this review, we solidified our findings structured around the themes.

## Results

### Characteristics

The majority of the articles (18/22) were qualitative, 3 papers used mixed methods (3/22) and 1 used quantitative methods (1/22), respectively. A total of 13 (13/22) studies were based on primary data collection, and the remaining nine (9/22) were descriptive or based on secondary data. Over half of the articles reported on findings from the African continent (14/22), alongside three from Latin America (3/22), three from Asia (3/22) and 2 with multi-sited locations (2/22). Almost half of the articles (10/22) had a full-paper focus on advocacy and activism, whereas the rest (12/22) discussed it in conjunction with the broader theme of the paper.

Our findings found that the terms advocacy and activism were generally undefined. No clear distinctions or differentiations were made between the terms advocacy and activism. The term ‘activism’ was only used as a term in conjunction with ‘advocacy’ and in papers that had a central focus. This was further complicated by the fact that there was no apparent cross-cutting understanding or cohesion around how the terms were understood and used.

**Figure 2.**
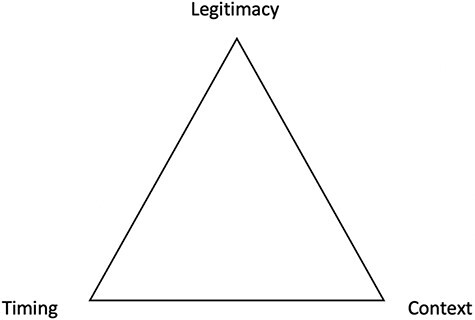
Model for mental health activism and advocacy analysis

We found three higher-level categories, under which we present our findings. These categories—legitimacy, context and timing—supported the creation of our framework (Figure 2), which we expand on in the Discussion section. Table 3 provides an overview of the themes. We suggest that our proposed framework can be used by researchers and practitioners as a starting point to develop a deeper understanding of advocacy and activism, considering the nebulous and undefined understanding surrounding them.

**Table 3.** Selected quotes for thematic categories and themes

Thematic category	Theme	Selected quote
Legitimacy	Internal legitimacy External legitimacy Negotiation of legitimacy	*Social capital was recognized as important. All subgroups agreed that the organization had done tremendous work increasing its social capital (bonding internally and bridging trust and respect with other groups and networks across society, especially within the disability rights movement), but that more remained to be achieved. We think social capital is essential if groups are to assume a leadership role in grassroots advocacy for change in mental health systems.* (Nickels *et al*., 2016, p.12) The Country Facilitators, as well as these graduates, are guided by priorities set by a wide coalition of stakeholders in the country, creating a unified message, with the legitimacy that comes from such a broad-based constituency. Indeed, the expectation from the leadership course participants is that they become informed advocates for mental health service development in their respective countries (Abdulmalik *et al*., 2014, p.5) *Additionally, stakeholders emphasized a need for research evidence that strengthens the business case for mental health care as planners consider value for money when distributing funds. Such evidence would give a strong case to advocacy campaigns*. (Ssebunnya *et al*., 2018, p.7)
Context	Direct context Broad context	*Currently, mental health care is characterized by a distinct lack of service options and imprecise estimates of need. In 2009, the WHO estimated that 2058 people received some form of formal mental health treatment, suggesting a 98% treatment gap for severe mental disorders using global estimates of prevalence. This treatment gap reflects a dearth of human resources; there are currently two psychiatrists, no psychologists and 20 psychiatric nurses in the country. There are limited resources for mental health in the public hospitals; no beds or transportation vehicles are allocated to mental health in the Bo district hospital. The limited mental health services available are provided primarily through the Sierra Leone Psychiatric Hospital and the City of Rest, a faith-based substance abuse/mental health inpatient setting in Freetown. These institutions have also experienced significant physical limitations, including unusable water and poor housing*. (Fitts *et al*., 2020, p. 658) *Another characteristic of the Argentine context is the cultural value attached to political participation. To participate and being an activist is something that really matters for Argentineans*. (Rosales *et al*., 2018, p.1360)
Timing	External timing Created timing	*South Africa is currently at the early stages of reviewing its national policy on mental health. This policy review presents an ideal opportunity to engage mental health care service users as informants in the policy process*. (Kleintjes *et al*., 2012, p.2273) It is now commonly recognized that effective advocacy has to be a central component of efforts directed at generating political will [14] for increased attention to mental health service improvement and scale-up as well as reducing the level of public stigmatization of the mentally ill [15–19]. Such advocacy presents access to mental health services as a fundamental human right [20, 21] and derives its strength from harnessing the commitment and energy of key stakeholders, including mental health professionals, policy makers, service users and families. (Abdulmalik *et al*., 2014, p.2)

### Legitimacy: an invisible driver shaping collective action

The need for, and pursuit of, legitimacy emerged across multiple papers. For a structured approach to legitimacy, we are informed by Lamb’s 2014 conceptualization of legitimacy. Roughly put, legitimacy denotes ‘worthiness to support’ and is broadly applicable (Lamb, 2014). The unit of analysis we use is ‘collective action’, which we refer to interchangeably as ‘engagement’. The findings indicated a general presence of internal legitimacy amongst advocate groups and efforts. This was expected, as phenomena such as social movements and activism usually have strong internal legitimacy because individuals are engaged in them as they believe in their goals. This is exemplified in various papers that describe the creation of social capital amongst advocacy groups—which is driven by legitimacy (Lamb, 2014). However, we also noted instances where internal legitimacy was lacking. For example, Abayneh *et al*. (2017) found that service users felt that it was the responsibility of government workers to enact change, hence they did not feel legitimated or confident to conceive ways in which they might contribute to system strengthening as advocates. Internal legitimacy was found to be lacking in instances where external stakeholders tried ‘recruiting’ advocates, and as such indicates advocacy efforts that were not initiated by advocates themselves. This represents situations where individuals are encouraged to become advocates as part of top-down strategies for system strengthening. One paper refers to this as utilitarian (Rose, 2018); another describes it as prescriptions of context-indifferent formulas put forward by initiatives from actors such as WHO (Montenegro and Cornish, 2019). This highlights the co-optation of the term ‘advocacy’, as it becomes employed strategically by external actors to further their own goals and shift responsibility—often serving top-down agendas. This co-optation was further emphasized as we found it to be void of any desire to change the status quo, which is central to our guiding definition of health activism. Ganesh and Zoller (2012) observed such practices across various activist movements, where discourse becomes manipulated and framed around superficial similarities to give the impression of collaboration, as a tactic of (productive) power.

External legitimacy—conferred by external actors such as wider communities and the state—emerged to be similarly important. Legitimacy is closely interlinked with stigma, and a lack thereof was seemingly fuelled by stigmatization. For example, some stakeholders perpetuated the idea that service users would not be capable of engaging in advocacy due to their symptoms, which served as a way to exclude their potential advocacy efforts (Irmansyah *et al*., 2020). By delegitimizing their abilities, the casting of such doubts narrows the scope of engagement before issues are even encountered. In a similar vein, the expertise of service users with lived experience was often delegitimized through accusations that they lack scientific knowledge, mental health literacy, or data and evidence to support the need for change. By establishing science as the sole valid form of evidence, their efforts became delegitimized. Findings suggest that a lack of legitimacy can be costly for advocactes and activists, both in terms of resources and time. For example, Ssebunnya *et al*. (2018) explains that the stigmatized nature of mental health and high turnaround of officials in Uganda pushes advocates to use most of their energy in renegotiating the importance of mental health with new officials. Hendler *et al*. (2016) noted similar challenges in Zimbabwe. Given the limited resources of activists, this can considerably impact the sustainability of their efforts.

However, our findings showed that legitimacy is not static and can be negotiated. A common pathway observed to gain legitimacy is the adoption of a generally legitimized professionalized profile. One study describes a project that recruits mental health leaders and advocates across West Africa to provide training to equip advocates with a particular skill set and knowledge base (Abdulmalik *et al*., 2014). In the grey literature, Mental Health Society Ghana similarly describes the provision of advocacy training to gain advocacy skills. However, this process homogenizes the pool of experts to fit a particular profile, casting doubts as to whether they are seen as experts in their own right, or whether they are perceived as experts by virtue of fitting the criteria of a particular legitimized required skill set. If the latter is true, this implies the need to live up to expectations and formulate advocacy goals in line with the associated ‘professional profile’ in order to retain legitimacy. Montenegro and Cornish (2019) highlighted that this can result in dual-framing advocacy, where seemingly contradictory positions and realities of advocacy co-exist and seek to find the balance between operating inside and outside the system.

### Context: shaping the goals and mode of advocacy

Context emerged as a key thematic area to discern how advocacy efforts come to life and evolve. The importance of context was explicitly stated in a number of studies. However, the importance of context was more often suggested implicitly, through inclusions of context interwoven in the studies. The proximal context—pertaining to (mental) health systems—most often contributed to determining concrete goals of advocacy and activist efforts. We identified goals such as change in policy or legislation, diversification of care options, implementation of community-based care and respect for human rights. Of course, health systems need to be understood in relation to the broader, distal context within which they are situated. For example, Kimangale *et al*. (2021) note the importance of historical context in order to understand care provision today, as coloniality remains pervasive. In the grey literature, we found this evidenced by Mariwala Health Initiative publication of the Queer Affirmative Counselling Practice resource book—a resource to guide mental health practitioners in adapting their practice in ways that are more directly informed by the distal context and incorporate greater awareness of broader queer and trans lived experiences in India.

We found that the distal context—including social, political, economic and historical context—was important to understand the mode in which advocacy and activism take place. Socio-political context was found to be particularly crucial, as it shapes how change might be pushed for and received. For example, Rosales and colleagues (2018) explain that civic participation is an important part of identity and belonging in Argentina, and that a desire for individuals to be politically and socially engaged is common, which facilitated engagement in mental health activism. Kleintjes *et al*. (2012) similarly noted the importance of participation in South Africa, particularly inspired by the model of the Treatment Action Campaign, which made it easier to engage in mental health advocacy and activism. On the flipside, Abayneh *et al*. (2017) noted a different politico-social context in Ethiopia, in which civic participation was uncommon, making it harder for advocates to navigate the space of advocacy. These examples illustrate the need to consider contexts far beyond resources and care provision to understand how advocacy and activist efforts take shape.

### Timing: creating critical junctures in advocacy and activist processes

Timing represents a critical juncture, which sees certain factors align to create significant and meaningful opportunities for activism which can further their efforts—through both external and created timing. External timing was most often driven by momentum, which helped advocates gain visibility (and legitimacy) as interest was sparked around mental health. Changes in policy and legislation are examples of momentum, which gives rise to timing that aligns with the ability of advocates to become engaged and leverage more power than they were usually able to. For example, Irmansyah *et al*. (2020) found that the legislative improvements, which arose when Indonesia joined the United Nations Human Rights Council and Security Council, provided a unique opportunity and timing for civic engagement for mental health. Timing, for example, allowed for opportunities to integrate mental health into broader discussions in response to significant events as a way to warrant the importance of mental health considerations beyond clinical settings. This was exemplified in the grey literature as the organization Kushinga set up the Cyclone Idai Project, because the response to the devastating aftermath of the cyclone in Zimbabwe lacked a mental health component. However, timing can also be fleeting or fragile. For example, Deshpande *et al*. (2013) and Mohamed (2015) noted that in Egypt and the Maldives respectively, the opportunities created through the momentum from drafting new mental health policies fell flat after changes in regimes that did not show the same energy for the continuation of these efforts. Additionally, we found that timing is not always external, but can also be created. Instead of becoming engaged by other stakeholders when there was momentum, advocates proactively created momentum, e.g. by borrowing energy from other (related) movements like civil rights or human rights movements. This is often a slower process that requires sustained efforts to culminate in the alignment of various factors to allow processes of change.

What emerged as the most important aspect of timing is that to understand a critical juncture we ought to consider the prior history and sequencing that led up to it—as opposed to merely looking at it as a snapshot in time. Montenegro and Cornish (2019) argue that recognizing the contingent temporality of activist processes, which cannot be understood as a mere linear progression, contextualizes our understanding of them and opens up opportunities for ‘analytic alternatives’, which move beyond analysis that is merely focused around failures or successes.

## Discussion

Our review aimed to critically explore the driving factors that shape efforts of mental health-related advocacy and activism in LMIC settings. This effort was informed by a desire to understand how advocacy and activism locate themselves in the rhetoric of GMH that purports the need to centre lived experiences and realities. We begin by presenting the conceptual model, which arises out of our analysis, and then suggest recommendations for future research.

The review identified a small, yet growing, body of literature on mental health activism and advocacy in LMIC settings. The main takeaway being that there is an extremely fragmented picture in regards to our understanding of mental health activism and advocacy. The fragmentation starts at the very foundation, given that there is no cohesion around terms and definitions. We argue that this lack of coherence has pushed research in superficial directions which barely touch the tip of the iceberg of advocacy and activism. As a result, most research focuses on the outcomes of advocacy—whether success or failure—as these form visible and measurable aspects. This leads to the omission of aspects that lie under the surface, such as processes, mechanisms, motivations and pathways, and it overshadows the core and essence of advocacy and activism, such as lived experience, by shifting the attention to other aspects.

We further suggest that the weakness around definitions has enabled the observed co-optation of the terms. By indicating (performative) support of advocacy, institutional actors may come to shape the discourse around advocacy through productive power. Given the absence of a generally accepted definition, their imposed direction becomes hard to contest. This results in a narrow framing of the perceived scope of action, pushing forward the idea that policy change and implementation are the main—and sometimes the only valid—goals of advocacy and activism. The narrow scope and macro-level focus creates a skewed image that serves to fit top-down agendas by virtue of the fact that advocacy is conceptualized in a manner that is more easily controlled. This is not uncommon in institutional settings, which strategically use discourse across sectors to manipulate terminology in order to frame and control the parameters of policy problems and solutions (Manzi, 2012).

The proposed model is intended to serve as a starting point towards deeper explorations and more nuanced understandings of mental health advocacy and activism. Allowing for deeper understanding can then create opportunities to live up to the promises of GMH to centre the voices of lived experience and community-centred solutions. This model helps to encourage interrogations around how processes unfold and evolve, in ways that attribute value to aspects beyond just a visible or measurable outcome. Although we recognize that there may be other aspects at play too, our findings suggest the use of three main dimensions to help structure and frame our approach: ‘legitimacy’, ‘context’ and ‘timing’. It is important to recognize the interrelatedness of these three categories, and that they rarely operate as discrete or separate categories. We note that they are all underpinned by power relations, which, although explored in our analysis, merit further in-depth attention in future studies.

The dimension of legitimacy, both internal and external, opens up possibilities of thinking about advocacy and activism in more encompassing ways by allowing critical exploration of multiple facets, as it is a defining factor in both processes and outcomes. For example, where internal legitimacy was lacking, it drew our attention to the co-optation of the term advocacy, as tensions became apparent through the lack of empowerment evidenced by service users. Moreover, considering legitimacy allows activism to be situated within a wider system through the comprehension of how others grant legitimacy and to whom. This enables integration of crucial considerations about power, as legitimacy is both a source of power as well as a constraint on it (Bexell, 2019). In the context of mental health, this is essential given that stigma—which remains prevalent—is considered a manifestation of power (Tyler and Slater, 2018). In light of this, we suggest that theories of social psychology, particularly that of social representations (Moscovici, 1988; 2001), can help our understanding of this dimension. Social representations contribute to the processes of the social construction of meaning. Consequently, the way social representations of mental health and associated advocacy and activism efforts are constructed—and form part of social knowledge—influences the categorization between legitimized or non-legitimized collective action, which serves as a social guide in terms of how these are to be reacted to. This then influences how advocacy or activism exists and presents itself. Our findings, supported by theory, show that social representations are at times negotiated in order to gain legitimacy (Howarth, 2001). This was exemplified by the professionalization of advocacy, an accepted identity for enacting higher-level changes, because it is associated with legitimized and reified knowledge systems. Trends of professionalization amongst civil society organizations have been noted across a wider body of literature, including international development (Srinivas, 2008; Gulrajani, 2011; Girei, 2023). Although legitimacy may not necessarily be desired or sought after, the presence or lack of legitimacy deeply shapes advocacy and activism in terms of how they are perceived and reacted to by others, thus inevitably shaping and influencing how they operate, create meaning and set goals.

The second dimension is context, including both proximal and distal context. Proximal context led more directly to the shaping of clearly defined and actionable goals such as policy change, diversification of care, implementation of community-based care and respect of human rights. We recognize that sometimes goals of advocacy may be more ‘abstract’ or ambiguous, such as fighting stigma, in which case they are rather influenced by the distal context. Whilst the distal context does contribute to influencing goals, it most importantly influences the mode in which advocacy and activism operate. The need to consider the contextual reality of health systems, as well as the wider context beyond it, is concordant with the literature on social movements for mental health in HIC settings (Crossley, 2006; Roa and Klugman, 2014). For example, the findings highlight how the socio-political context contributes to the social acceptance (or lack therefore) of civic engagement, thus requiring adaptation of the ways in which people resist and contest an unfavourable status quo. This leads back to the importance of social representation, and how positive social representations held by others enable and shape possibilities of engagement.

Timing refers to a critical juncture where factors align to create an opportunity for engagement that might not have been possible previously. This does not imply that advocacy cannot happen otherwise (in a ‘business as usual’ situation), but that these junctures may lead to a focus shift or catapult outcomes through opportunities that have arisen from a change in the parameters of possibilities. External timing is also often driven by momentum, e.g. from policy cycles, creating situations wherein the parameters of engagement are (temporarily) widened and allow for strategic involvement. Created timing highlighted how, through negotiations of their space, advocates themselves can create new openings for engagement. This can be observed in the literature with the example of the use of strategic litigations as advocacy tools, as litigations put forward cases of public interest that may have a broader impact on systemic structures beyond the individual plaintiffs (Roa and Klugman, 2014). This aspect also highlights the agency of advocates and activists, which often goes amiss amongst discussions that focus on environmental constrictions. The importance of timing has been put forward by political scientists to explain why and how institutional changes take place, arguing that we must consider the ‘when’ alongside the ‘what’. This provides a holistic view that understands that the past and its sequencing matter to understanding the present (Pierson, 2000; 2011). Our model suggests that timing, as a critical juncture understood through the timeline that precedes it, contributes to our understanding of advocacy and activism. This is concordant with Kingdon’s (1984) windows of opportunity, though we believe these critical junctures to be relevant for milestones beyond institutional ones such as agenda-setting or policy-making. As such, this dimension helps to introduce the analytical importance of advocacy and activist processes beyond mere outcomes, as the process is crucial to the understanding of timing.

### Suggestions

In light of our findings, we provide the following suggestions for future research.

Our first suggestion relates to the findings that highlight the co-optation of the term ‘advocacy’ by institutional discourses. The term is becoming used as a form of scapegoat and source of blame when governmental efforts fail, as well as a discursive tool for shifting responsibility and work onto advocates. We suggest—where possible—to clearly differentiate approaches that seek to include and centre bottom-up perspectives and voices. To do so, we recommend shifting towards using the term ‘activism’ as a means of signalling action that seeks to embed marginalized voices and emancipatory action. This helps to differentiate from institutional conceptualizations of ‘advocacy’, where top-down voices decide when and where advocacy is valid, with pre-determined goals imposed on advocates. Both the power and the danger of co-optation form part of ongoing discussion amongst mental health activists in HIC settings, but remain under-addressed in the literature in LMICs. Of course, a change in terminology mostly cures the symptoms of a much deeper-rooted problem, however, it holds symbolic power and is a first step in a better direction.

Secondly, there is a need for new ways to approach the study of advocacy and activism in order to bring dynamism and diversity in terms of research. We suggest shifting the focus away from a dichotomous attention on failures vs achievements, towards a more whole-encompassing approach that also considers the ‘process’ of activism. Focusing on ‘process’ might yield useful findings tying into these existing bodies of literature. Furthermore, those understandings might also enable us to better understand the current aspects that are highlighted in the literature—failures and achievements—in more meaningful ways.

Lastly, we suggest the need to diversify ‘how’ we research mental health advocacy and activism. Our review found little variety in the methods used. Most papers were qualitative and descriptive in nature. Where primary data is included, it is largely based on (semi-structured) interviews and occasional focus-group discussions. These methods hold merit; however, we limit the scope and possibilities for knowledge by omitting other methods. Whilst our suggestion is broad, we particularly underline the need for more ethnographic work in this space. As Jain and Orr (2016) point out, ethnography remains vastly underutilized in global health as a whole. Ethnography can also be a valuable approach in relation to the previous suggestion on exploring advocacy processes. It would enable a move beyond the current trend of looking at mental health activism from a bird’s eye view perspective, towards deeper understandings of everyday pathways of resistance and change, and the implications and experiences of such engagement on communities.

### Limitations

First, we acknowledge that many advocacy or activist efforts might not explicitly label themselves as such, even if they are inherently activist as per our guiding definition. Therefore, literature that did not use related labels might have been missed in the search and screening process. Secondly, we were constrained by the small number of papers with a central focus on advocacy, which may have limited the level of depth in analysis, as well as the fact that the majority of the studies were qualitative in nature, and hence not necessarily generalizable to wider groups. Lastly, the search was restricted to English language, which might have excluded relevant research published in other languages, particularly as many of the countries included do not have English as their primary language.

## Supplementary Material

czae016_Supp

## Reflexivity statement

All authors are female and span multiple levels of seniority. All authors have experience, on varying levels, of mental health-related research in the Global South. Three of the authors have extensive experience in conducting qualitative fieldwork in various countries of the Global South, including Zimbabwe, Malawi, Colombia, Afghanistan, Peru, South Africa and Samoa.
